# Antagonistic Effects of CAPE (a Component of Propolis) on the Cytotoxicity and Genotoxicity of Irinotecan and SN38 in Human Gastrointestinal Cancer Cells In Vitro

**DOI:** 10.3390/molecules25030658

**Published:** 2020-02-04

**Authors:** Gabriela Gajek, Beata Marciniak, Jarosław Lewkowski, Renata Kontek

**Affiliations:** 1 Laboratory of Cytogenetics, Institute of Experimental Biology, Faculty of Biology and Environmental Protection, University of Lodz, 12/16 Banacha St., 90-237 Lodz, Poland; beata.marciniak@biol.uni.lodz.pl (B.M.); renata.kontek@biol.uni.lodz.pl (R.K.); 2 Department of Organic Chemistry, Faculty of Chemistry, University of Lodz, 12 Tamka St., 91-403 Lodz, Poland; jaroslaw.lewkowski@chemia.uni.lodz.pl

**Keywords:** CAPE, irinotecan, SN38, cytotoxicity, genotoxicity, oxidative stress

## Abstract

The incidence of gastrointestinal cancers is increasing every year. Irinotecan (CPT-11), a drug used in the treatment of colorectal cancer and gastric cancer, is metabolized by carboxylesterases to an active metabolite, SN-38, which is more cytotoxic. CAPE (caffeic acid phenethyl ester) is an active component of propolis, which has a high antibacterial, antiviral, and antineoplastic potential. This study analyses the impact of CAPE on the cytotoxic (MTT assay), genotoxic (comet assay) and proapoptotic (caspase-3/7 activity) potential of irinotecan and its metabolite SN-38 in cultures of gastrointestinal neoplastic cells (HCT116, HT29, AGS). Cytotoxicity and genotoxicity activities of these compounds were carried out in comparison with human peripheral blood lymphocytes (PBLs) in vitro. The antioxidant potential of CAPE was investigated in relation H_2_O_2_-induced oxidative stress in the both neoplastic cells and PBLs. CAPE expressed cytotoxic, genotoxic, and pro-apoptotic activity against AGS, HCT116, and HT29 tumor cells. CAPE, in the presence of different concentrations of irinotecan or SN38, decreased the cytotoxicity, genotoxicity, and pro-apoptotic activity in these cell lines, but it has no such action on normal human peripheral blood lymphocytes.

## 1. Introduction

Gastric and colorectal cancers are the third and fourth cause of death worldwide and concern ~1.5 million people. Irinotecan (CPT-11), a semi-synthetic derivative of the naturally occurring alkaloid camptothecin, is a medication commonly used in the management of gastrointestinal cancers [1]. Its mechanism of action involves inhibition of DNA topoisomerase I, thereby stabilizing complexes during DNA replication, and leading to cell death [2]. CPT-11 is converted by carboxyloesterases in vivo to its much more active metabolite, 7-ethyl-10-hydroxy camptothecin (SN38) [3]. The cytotoxic effects of CPT-11 and SN38 are time-dependent and specific for the S-phase of the cell cycle [4].

A growing number of neoplastic patients take dietary supplements to minimize the side effects of chemotherapeutic agents, and thereby facilitate the therapeutic effect. Supplementing the everyday diet with vitamins or other exogenous substances is also aimed at improving the efficacy of chemotherapy, which does not always have the desired therapeutic effect. There are two contradictory hypotheses concerning the presence of antioxidant dietary supplements taken by patients in the course of chemotherapy. The administration of supplements may significantly improve the therapeutic effect, protect the healthy tissues against the toxic activity of the medications, and reduce the side effects [5,6,7]. On the other hand, strong antioxidants in a patients’ diet may decrease the toxic effect of the medications used in neoplastic cells, thus negatively affecting the efficacy of chemotherapy [8,9].

Propolis (also known as bee glue), with its wide spectrum of medicinal properties, is one of the commonly used dietary supplements that is taken by both ill and healthy people. The substance is of natural origin, collected by bees from parts of flowers, mainly the buds, but also from tree or bush resin [10]. Bee glue has proven antifungal [11], antibacterial [12,13], anti-inflammatory [14], and antioxidant properties [15,16]. Owing to its therapeutic properties, it has been used in natural medicine for many years worldwide, and it is nowadays available as a dietary supplement and functional food component.

**Figure 1:**
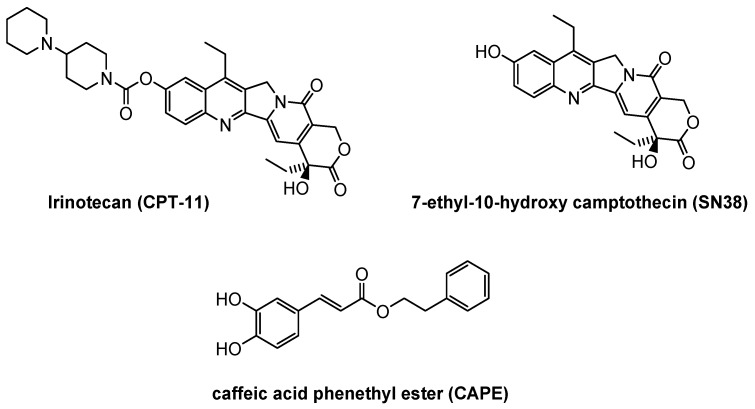
The structural formula of compounds studied in this work.

One of the main, biologically active components of propolis is caffeic acid phenethyl ester (CAPE). This compound is part of the group of polyphenols, and hydroxyl groups of the catechol ring (Figure 1) are largely responsible for its extraordinary biological properties [17]. The compound is also a well-known and specific NF-κB (nuclear transcription factor activity) inhibitor, which is engaged in many cell processes, e.g., apoptosis, proliferation, differentiation, inflammation and immune system responses [18,19]. The literature is more frequently reporting on the antineoplastic activity of CAPE on many cancer models in vitro and in vivo [20,21,22], with particular regard to its antiproliferative, cytotoxic and proapoptotic properties against the neoplastic line cells [17,23,24]. This compound is characterized by selective cytotoxic activity against neoplastic line cells rather than normal cells [25].

CAPE as a biologically active propolis component may be an effective chemotherapeutic agent. However, the question arises, whether it can be safely administered together with many of the conventional anticancer agents? To answer this question, we have used three cancer cell lines of the gastrointestinal tract—AGS (human gastric adenocarcinoma), HCT116 (human colorectal carcinoma), HT29 (human colorectal adenocarcinoma)—as well as human peripheral blood lymphocytes (PBLs) as a normal cell control, with the aim of analyzing the biomodulation potential of CAPE on the cytotoxic and genotoxic activity of CPT-11 and SN38 on them.

## 2. Results

### 2.1. Cytotoxicity

The cytotoxicity was assessed by means of a MTT test and evaluated by IC_50_ values, concentration of drug that cause 50% growth inhibition. The obtained IC_50_ values were summarized in Table 1.

**Figure 2:**
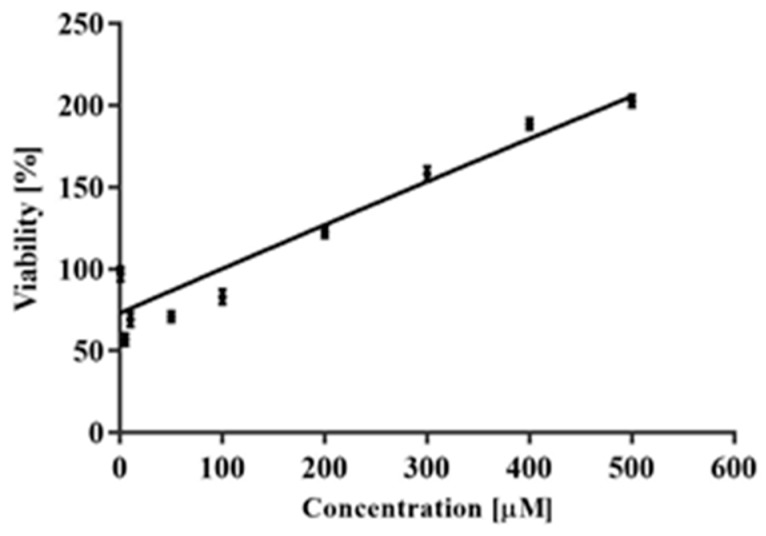
The effect of CAPE on PBLs growth after 72 h incubation evaluated by MTT assay (the mean ± SEM).

After 72 h incubation with CAPE, CPT–11 and SN38 reduced the viability of neoplastic cells along with an increase in the concentration of these analyzed compounds. The highest cytotoxic activity occurred by incubation of the cells with SN38 at 0.25–30 µM in all the analyzed experimental series, whereas the lowest cytotoxicity occurred after incubation with CAPE at 0.5–500 µM. MTT assay was used to determine the appropriate values of IC_50_, for CAPE, CPT-11 and SN38, which were used in subsequent experimental series involving incubation simultaneously of CAPE with CPT-11 and CAPE with SN38 (Table 1). Among the cancer cell lines, HT29 cells were the most sensitive to SN38 activity, with an IC_50_ value of 0.3 μM. At the same time, these cells were the least sensitive to CPT-11 and CAPE, IC_50_ values being 8.5 and 24 μM, respectively. HCT116 cells were the most sensitive to CPT–11 activity. Similar IC_50_ values were obtained for HCT116 and AGS cells after CAPE treatment (8 μM). Noteworthy, after treating AGS cells with CAPE and CPT-11, the IC_50_ values were identical. In turn, the values of IC_50_ obtained following exposure of PBLs to CPT-11 and SN38 were significantly higher than those for neoplastic lines, being 200 and 16 µM, respectively. PBL incubation with CAPE did not decrease the lifespan of cells in relation to the negative control, and therefore determining the value of IC_50_ became impossible (Figure 2).

#### Antagonistic Effect of CAPE on CPT-11 and SN38

**Figure 3:**
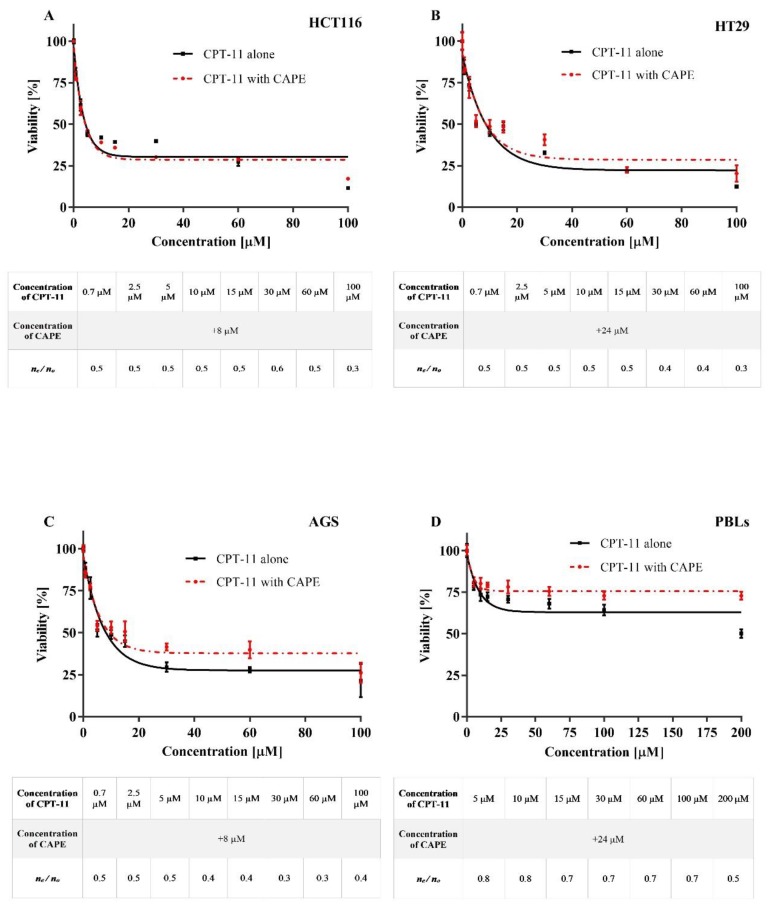
The effect of CPT-11 and co-treatment CAPE + CPT-11 on HCT116 cells (**A**), HT29 cells (**B**), AGS cells (**C**) and PBLs (**D**) growth after 72 h incubation evaluated by MTT assay (the mean ± SEM). Treatment of CAPE and CPT-11 simultaneously shows antagonistic effect on cytotoxicity of HT29, HCT116, AGS cells and PBLs. The figure under the graph show the ratio of expected cell number (n_e_)/observed cell number (n_o_). For example, treatment of 8 µM of CAPE or 15 µM irinotecan decreases cell number of HT29 to 50.00% and 48.00%, respectively, compared to the control (no treatment). The expected cell number of treatment combining 5 µM of CAPE and 15 µM irinotecan is 0.50 × 0.48 = 24%. The observed cell number is 48% compared to the control. The ratio is 0.24/0.48 = 0.5. Factor < 1 represents antagonistic effect CAPE on CPT–11.

**Figure 4:**
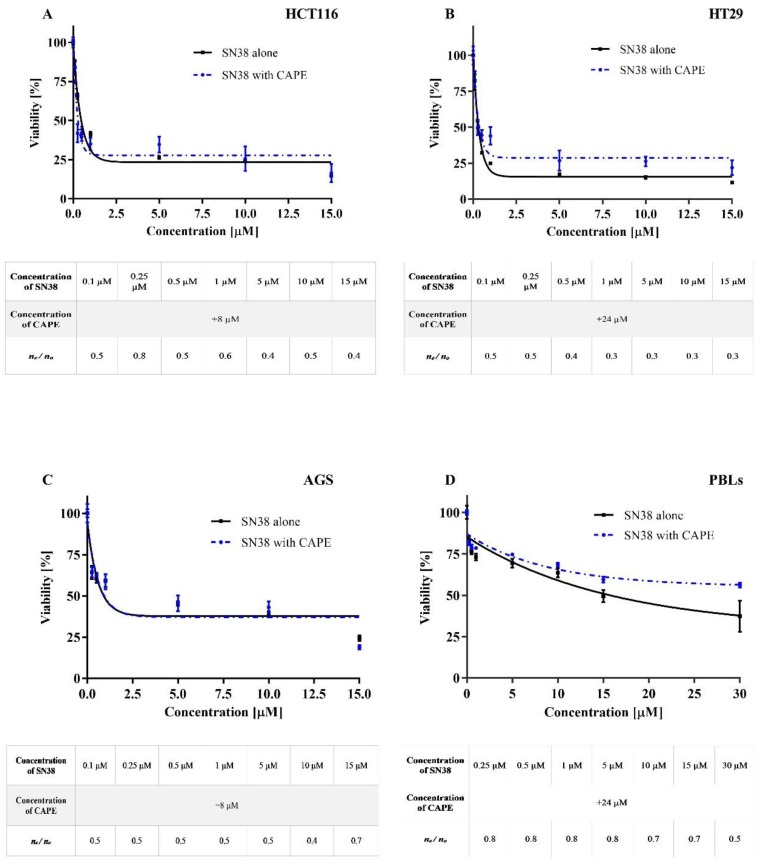
The effect of SN38 and co-treatment CAPE+SN38 on HCT116 cells (**A**), HT29 cells (**B**), AGS cells (**C**) and PBLs (**D**) growth after 72 h incubation evaluated by MTT assay (the mean ± SEM) Treatment of CAPE and SN38 simultaneously shows antagonistic effect on cytotoxicity of HT29, HCT116, AGS cells and PBLs. The figure under the graph show the ratio of expected cell number/observed cell number. Factor < 1 represents antagonistic effect CAPE on SN38.

Simultaneous incubation with the compounds under test was used to assess the effect of CAPE on cytotoxicity induced by CPT-11 and SN38 in the HT29, HCT116, AGS and PBLs cells. CPT-11 was added to the medium at 5, 10, 15, 30, 60, and 100 μM, whereas for SN38 it was 0.25, 0.5, 1, 5, 10, and 15 μM. For each concentration of CPT-11 and SN38, CAPE was added at the concentration equal to the IC_50_ determined for each of the tested cell lines. The results obtained for experimental series CAPE + CPT-11 and CAPE+SN38 are shown in Figure 3 and Figure 4, respectively.

In the case of the HT29 line cells, co-incubation with CAPE and CPT-11 resulted in increased cytotoxicity of CPT-11 compared to CPT-11 alone. The same modus operandi for the two remaining cancer cell lines (HCT116 and AGS) led to opposite results, as simultaneous action of CAPE and CPT-11 (at >IC_50_) in the culture medium decreased the cytotoxic activity of irinotecan, and thus the lifespan of the cells increased (Figure 3). Interestingly CAPE had no effect on the change in the IC_50_ value specified for neoplastic cells subjected to CPT-11 (Table 1). The results obtained with PBLs showed that CAPE increased their lifespan up to ~75% when incubated with irinotecan, and even at high concentrations of the drug. Based on the results obtained, we have determined the antagonistic effect of CAPE on the cytotoxic activity of irinotecan for every concentration of the medication under analysis.

In the second experimental series, CAPE was added to the culture medium of the cells together with SN38 for 72 h. The results obtained for the HT29 cell line are of interest because CAPE decreased the IC_50_ value for SN38, so its effect decreased its cytotoxic (Figure 4). Regarding HCT116 and AGS cells, the ester had limited (HCT116) or no effect (AGS) on change in the IC_50_ of SN38. The addition of CAPE along with SN38 increased the lifespan of HCT116 line cells compared to SN38 alone. With AGS cells, there were no significant differences between the series with or without CAPE. The survival rate of PBLs exposed to CAPE and SN38 increased compared to SN38 given alone. As with irinotecan, there was an antagonistic effect of CAPE on the cytotoxicity of SN38 at all the concentrations used (Figure 4).

### 2.2. Caspase 3/7 Activity

The effect of test compounds on caspase-3/7 activity in adherent neoplastic cells was assessed after 24 h and 72 h incubation using ApoTox–Glo™ Triplex Assay. The concentrations of tested compounds correspond to the IC_50_ values determined for each compound in MTT assay.

**Figure 5:**
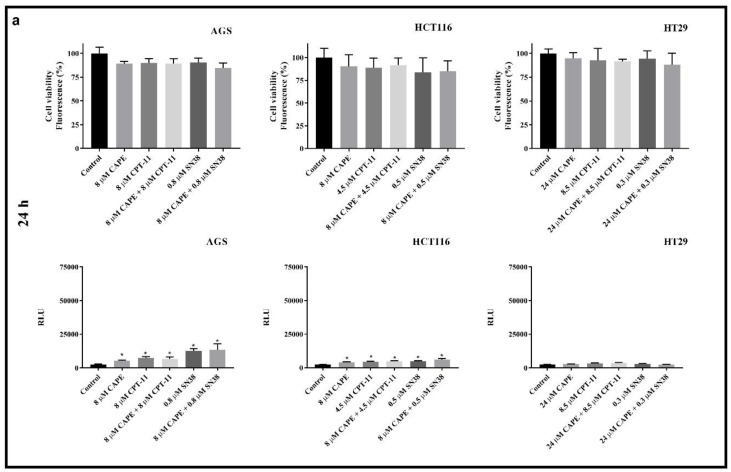

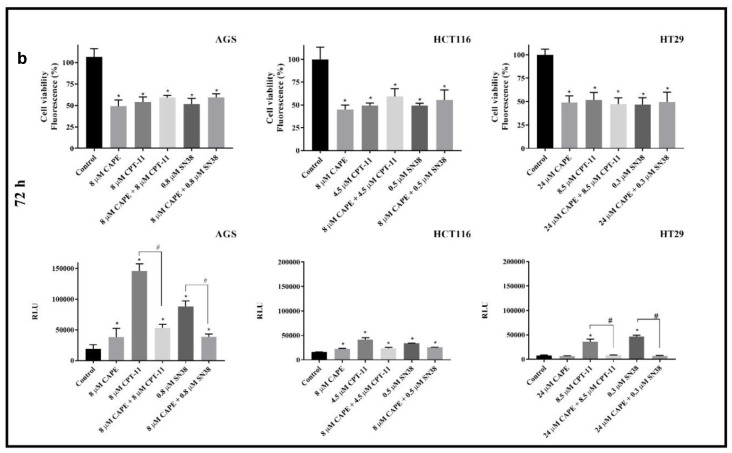
Viability and caspase-3/7 activity after 24 h (**a**) and 72 h (**b**) of exposure on CAPE, CPT-11, CAPE + CPT-11, SN38 and CAPE + SN38 in AGS, HCT116 and HT29 cell line (mean ± S.E.M). * *p* < 0.05 as compared with control (untreated) cells; ^#^ *p* < 0.05 as compared with cells treated drug.

The cell viability observed after 24-h and 72-h incubation with the tested compounds was measured considering activity of intracellular proteases. The integrity of cell membranes directly corresponds to number of viable cells in samples. Furthermore, the activity of apoptotic proteases—caspase 3/7 was assessed as a main objective. There was no statistically significant decrease in viability after 24 h of incubation with the tested compounds on all the cell lines (Figure 5a). At the same time, in the AGS and HCT116 was obtained statistically significant increase in the activity of caspase-3/7 in all the tested series. Such an effect was not seen with the HT29 cell line. The differences in caspase 3/7 activity in samples treated with CPT-11/SN38 alone and with addition of CAPE (CPT-11/SN38 and CAPE together) indicate antagonistic effects between the tested compounds.

Figure 5b shows cell viability only as IC_50_ values. This confirms the results obtained in MTT assay. As previously mentioned in the manuscript, CAPE had no effect on the change of the IC_50_ values of neoplastic cells subjected to CPT-11 or SN38 (the exception was the HCT116 cell line treated simultaneously with CAPE and SN38). After 72 h exposure (Figure 5b), the AGS cell line had the highest activity of caspases-3/7, but it decreased after treatment with CAPE + CPT-11 and CAPE + SN38 compared to CPT-11 alone and SN-38 alone. A similar effect was seen with the HT29 cell line, but at a much lower level. In the HCT116 line after 72 h incubation, caspase-3/7 activity increased compared to 24 h incubation. No significant differences were noted between the series with the drug alone (CPT-11, SN38) and the drug + CAPE.

### 2.3. DNA Damage

#### 2.3.1. Genotoxicity of CAPE

**Figure 6:**
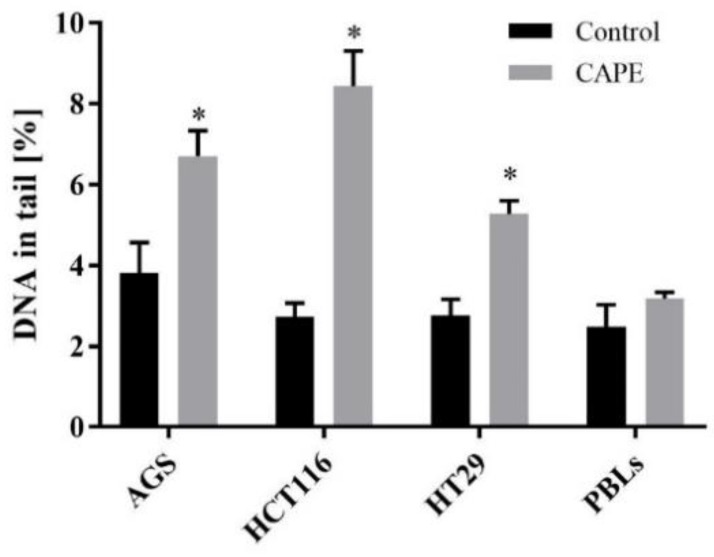
DNA damage in AGS, HCT116, HT29 cells and PBLs treated with CAPE for 24 h was measured by monitoring the percentage of DNA in the comet tail using alkaline version of comet assay (mean ± S.E.M). * *p* < 0.05 as compared with control (untreated) cells.

The level of DNA damage was measured following exposure of all the cell lines to CAPE, at 8 µM as regards AGS and HCT116, and 24 µM as regards the HT29 line (IC_50_ values designated in the MTT assay). In the case of lymphocytes, the highest concentration (of all those specified for neoplastic cells) was 24 µM. Cells were incubated with CAPE for 24 h, the results being shown in Figure 6.

The percentage DNA content in comet tails in all the experimental series did not exceed 10%. The most sensitive cells to CAPE were the HCT116 line, where DNA damage was up to 8.44% (Figure 6). Less DNA damage occurred in AGS and HT29 cells, for which the values were 6.71 and 5.58%, respectively. The lowest percentage of DNA damage occurred in PBL, at 3.13% (Figure 6). Data for all the neoplastic cells were significantly different from the negative control.

#### 2.3.2. Genotoxicity CAPE + CPT-11 and CAPE + SN38

**Figure 7:**
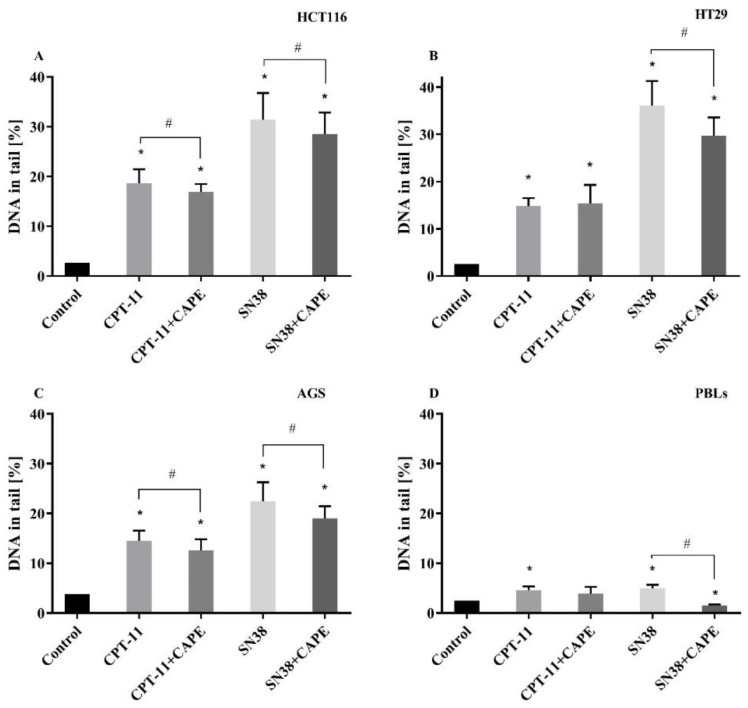
DNA damage measured in HCT116 (**A**), HT29 (**B**), AGS (**C**) and PBLs (**D**) as the percentage of DNA in the comet tail in the alkaline version of comet assay incubated 24 h with CAPE in the presence of CPT-11 or SN38 (mean ± S.E.M). * *p* < 0.05 as compared with control (untreated) cells; ^#^ *p* < 0.05 as compared with cells treated drug.

To determine the effect of CAPE on the genotoxic activity of CPT-11 and SN38 on AGS, HCT116 and HT29 and PBLs, they were incubated with it for 24 h at the same time as irinotecan or SN38 treatment at concentrations equal to IC_50_ specified for these particular compounds as regards the cells under treatment. Data for HCT116, AGS and PBLs showed that CAPE slightly decreased the genotoxic activity of CPT-11 and SN38 (Figure 7). In regard to HT29 cells, CPT-11 with CAPE increased genotoxicity more than CPT-11 alone, but the difference did not reach statistic insignificance. The biggest decrease in DNA% with SN38 + CAPE in comparison with SN38 alone occurred with HT29 cells.

### 2.4. Intracellular ROS Determined by H_2_-DCFDA

**Figure 8:**
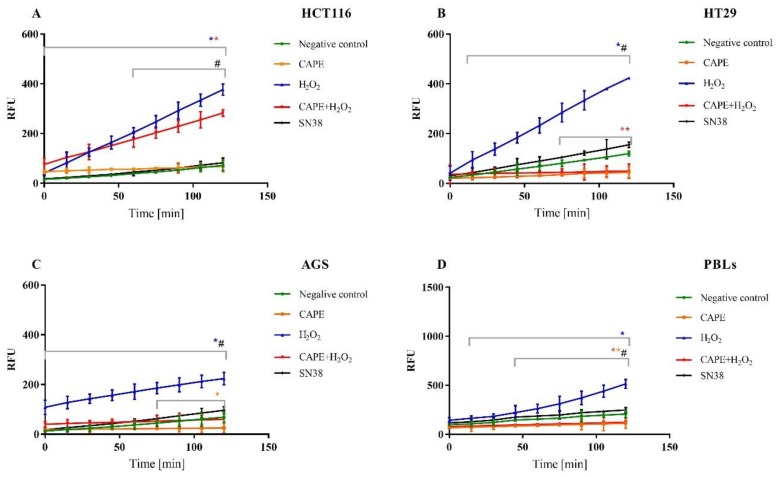
ROS generation in HCT116 (**A**), HT29 (**B**), AGS (**C**) cells and PBLs (**D**). The series with the preincubation with CAPE. The concentration of CAPE used in experiment was the IC_50_ value determined for each cancer cell line and for PBLs were used the highest concentration from all designated. * *p* < 0.05 as compared with control (untreated) cells; ^#^ *p* < 0.05 as compared cells treated H_2_O_2_ with cells treated CAPE + H_2_O_2_.

**Figure 9:**
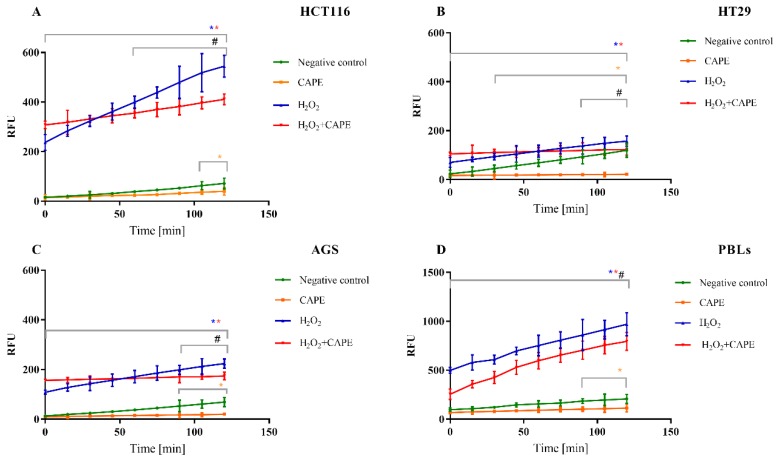
ROS generation in HCT116 (**A**), HT29 (**B**), AGS (**C**) cells and PBLs (**D**). The series with the preincubation with H_2_O_2_. The concentration of CAPE used in experiment was the IC_50_ value determined for each cancer cell line and for PBLs were used the highest concentration from all designated. * *p* < 0.05 as compared with control (untreated) cells; ^#^ *p* < 0.05 as compared cells treated H_2_O_2_ with cells treated H_2_O_2_ + CAPE.

The concentrations of SN38 used in our study (IC_50_ values determined for each cell line) did not induce a statistically significant increase in ROS compared to the negative control. Considering these findings, we decided to assess antioxidant activity of CAPE and compare it with H_2_O_2_-treated controls. Two experimental series were carried out: the first involved a 30 min preincubation with 2 mM H_2_O_2_, which was used as the oxidative stress inducer. Subsequently, CAPE was added at the time measurement began (at a concentration equal to IC_50_ designated for the particular cells). In the second series, CAPE was added 30 min before the measurement and subsequently 2 mM H_2_O_2_ was added. The negative control was used in each experimental series (PBLs), as well as positive control with CAPE (at the IC_50_ values specified for every neoplastic line) and with 2 mM H_2_O_2_ added, depending on the experimental series, 30 min before the measurement or during the course of measurement. Intracellular ROS was measured using the molecular probe H_2_-DCFDA. The results indicated that CAPE decreases the ROS induced by hydrogen peroxide for both experimental series in neoplastic cells as well as PBLs (Figure 8 and Figure 9).

The results were improved by adding CAPE 30 min before the oxidative stress inducer. The most effective reduction of reactive oxygen species was seen in HT29 cells. In both neoplastic cells and PBLs exposed to CAPE (at a concentration amounting to IC_50_ for particular lines and the highest of the designated ones for lymphocytes), it was noteworthy that the level of ROS was lower than in the negative control.

## 3. Discussion

There sufficient scientific evidence proving that healthy people taking antioxidants have reduced levels of ROS in their cells [26,27,28]. However, the effect of using dietary supplements with antioxidant properties during the course of antineoplastic therapy remains unknown. Some research shows that antioxidants may reduce the side effects of chemotherapy without affecting the drug action taken at the same time, yet other data suggest that supplements interfere negatively with chemotherapy and radiotherapy, i.e., reduce their efficacy [29,30]. The taking of antioxidant dietary supplements by cancer patients is becoming increasingly common, which makes research on the effects of supplements on the action of chemotherapeutic agents to be particularly important.

A characteristic feature of compounds used in anticancer therapy is their significant toxicity for neoplastic cells without damaging normal cells, i.e., their specificity. Our findings indicate that CAPE decreases the lifespan of cultured neoplastic cells. Moreover, the IC_50_ values of CAPE for the neoplastic lines used were significantly similar to the IC_50_ values determined for irinotecan – commonly used in anticancer therapy. CAPE is highly cytotoxic for AGS and HCT116 cell lines, with an IC_50_ of 8 µM. Lower cytotoxicity was seen with the HT29 line, for which the indicated IC_50_ was 24 µM. Many reports confirm the cytotoxic nature of CAPE [31,32,33], which are concordant with our results. Moreover, our findings concerning the cytotoxicity of CPT-11 and SN38 have been confirmed by others [34]. We also found that CAPE is non-cytotoxic as regards PBLs (seen as normal cells). Therefore, we have confirmed the preferential toxicity of this compound previously reported by Grunberger et al. [25]. Studies on drugs that reduce intracellular levels of free radicals have shown that they can induce an increase in PBLs [35]. Campbell et al. described the effect of vitamin C on in vitro culture of in vivo activated mouse T cells. While more than 70% apoptotic cells were found in cultures without vitamin C, the addition of vitamin C (450 µM) decreased apoptosis by one-third and induced more proliferation was seen compared to cultures without vitamin C [36]. Other study has shown that compared to the placebo group, an increase in T cell proliferation was seen in the vitamin C-supplemented group [37]. Similar results were obtained by Chen et al. (2015) who found edaravone enhanced cell survival at concentrations ranging from 10 to 100 μM in a dose-dependent manner at 24 h post-irradiation [38].

Co-incubation with CAPE and CPT-11 or SN38 at the concentrations used herein had no effect on IC_50_ values of gastrointestinal cancer cell lines treated with CPT-11 and SN38 as regards the series without CAPE. The results from series CAPE+SN38 with the HT29 line were an exception; in this case, the combined treatment of CAPE with SN38 increased the cytotoxicity of SN38 compared to treatment with SN38 alone. Analysis of the results for the range of concentrations over the designated IC_50_ values only for CPT-11 and the experimental series CAPE+CPT-11 led to a decrease in the cytotoxic potential of the medication in the HT29 and AGS cell lines, whereas CAPE treatment of the HCT116 line resulted in an increase in the cytotoxicity of CPT-11. Comparable results were obtained in the case of the experimental series CAPE+SN38, where CAPE decreased slightly the toxicity of SN38 operation in HT29 cells, HCT116 cells and PBLs. In the AGS line cells, CAPE given with SN38 did not significantly change the cytotoxic activity of SN38. The analysis showed that CAPE antagonises the cytotoxic potential of irinotecan and SN38. Similar studies were carried out by Lin et al. [39], who tested the modulatory effect of CAPE in relation to the chemotherapeutical activity of etoposide, paclitaxel, vinblastine, mitoxantrone and estramustine on prostate cancer cells (PC-3). CAPE had an antagonistic effect on the anti-proliferative activity of etoposide and mitoxantrone, whereas its effect on the activity of other medications was synergistic. Tolba et al. [40] have also reported synergistic activity of CAPE with the chemotherapeutic activity of paclitaxel and docetaxel in PC-3 cells. The discrepancy between our findings and these reports may result from different mechanisms of anticancer activity of the drugs used and hence their different interactions with CAPE. A common feature of drugs for which CAPE shows an antagonistic activity, namely etoposide, mitoxantrone, CPT-11, and SN38, is their impact on topoisomerase I or II. This property may explain the differences in the results.

Our investigation has proved the protective activity of CAPE on normal PBLs treated with the CPT-11 and SN38. The protective action of CAPE has also been proven by Fadillioglu et al. [41], who administered CAPE with a view to reducing the cardiotoxicity induced by doxorubicin in rats. Similar findings were obtained by Ozen et al. [42], who reported that the preventive use of CAPE protects kidneys from cisplatin-induced toxicity. Nephrotoxicity is not the only side effect of cisplatin, as it can also cause ototoxicity. Kizilay et al. [43] showed that the prophylactic use of CAPE in cisplatin therapy prevents deterioration of hearing in rats. Armagan et al. [44] proved the protective activity of CAPE against methotrexate-induced oxidative stress in the testicular cells of rats.

Studies on the ability of CAPE to induce apoptosis in cancer cells have shown that it induces caspase-3/7 activity in AGS and HCT116 after 24 h incubation. These results correlate with those of Yu et al. [45], which demonstrated the ability of CAPE activate caspase-3 and induce apoptosis in YD15, HSC-4 and HN22 cells. Similar results were obtained by Kabała-Dzik et al. [46] and Dziedzic et al. [47]; CAPE successfully induced apoptosis after 24 h incubation in MDA-MB-231 and NHSCC cell lines. In our work, HT29 was the most resistant to CAPE, which may be due to a mutation in the p53 gene affecting their resistance to apoptosis. On the other hand, Beauregard et al. [48] suggested that CAPE mediates the induction of apoptosis via a p53-independent pathway in MB-231 cells. Simultaneous incubation of the drug and CAPE decreased caspase-3/7 activity induced by CPT-11 and SN38 after 72 h.

Analysis of the degree of DNA migration of neoplastic cells of the gastrointestinal tract (AGS, HCT116 and HT29) using the comet assay indicated that CAPE can be characterized by its statistically significant genotoxicity. Our findings are confirmed by Watabe et al. [49], who proved that CAPE induces DNA fragmentation in PC-3 cells after 24 h incubation at a level similar to the experimental conditions we used. However, the results of CAPE co-incubation with the CPT-11 or SN38 indicated that CAPE decreased the genotoxic activity of both these compounds in all the cell types analyzed. The only exception was the HT29 cell line, where CAPE given along with CPT-11 increased genotoxicity compared to the series with CPT-11 alone, but the differences was not significant statistically. Yilmaz et al. [50] analyzed rat bone marrow cells to see the impact of CAPE on cisplatin-induced chromosomal aberrations. CAPE induced a statistically significant decrease in the number of abnormal metaphases and chromosomal aberrations compared to the absence of CAPE. They also showed that CAPE is protective in the bone marrow cells subjected to cisplatin treatment owing to its antioxidant activity.

The SN38 concentrations we used (IC_50_ values determined for each cell line) did not induce a statistically significant increase in ROS compared to the negative control. Similar results for concentrations below 1 µM SN38 were obtained by Santoro et al., 2015 [51]. This can be attributed and explained by the irinotecan’s mechanisms of action on cancer cells. Rapidly proliferating cancer cells accumulate DNA double-strand breaks resulting mainly from topoisomerase I inhibition upon exposure to irinotecan/SN38 [52]. Considering these findings, we decided to assess antioxidant activity of CAPE and compare it with H_2_O_2_ - treated controls. In this report, we have confirmed the antioxidant activity of CAPE in AGS, HCT116 and HT29, as also PBLs. Our research has shown that CAPE caused a statistically significant reduction in the level of ROS in cancer cells even compared to the negative control, this may be the reason for the antagonistic effect of CAPE in relation to the observed cytotoxic and genotoxic activity of CPT-11 and SN38. We have shown that CAPE added before the oxidative stress inducer gave better results than its addition after introducing 2mM H_2_O_2_. This might result from its ability to inhibit NF-κB, which is important in inducing oxidative stress. Chen et al. [53] have reported similar findings regarding the protective properties of CAPE against H_2_O_2_-induced death using the LDH assay in 661W mouse cells, i.e., CAPE influences inhibition of cell death in a dose-dependent manner. Sun et al. [54] used rat cardiomyocytes cells to follow the action of 2 mM H_2_O_2_; CAPE was protective, increasing the lifespan of the cells under oxidative stress. The antioxidant properties of CAPE have also been confirmed by Chen et al. [55], who found that in peripheral blood mononuclear cells CAPE inhibits xanthine oxidase, which, under physiological conditions, is the source of superoxide anions with the ability to inhibit lipid peroxidation. Moreover, studies suggested that the presence of CAPE in propolis is the main factor determining its antioxidant properties [15].

CAPE has strong anticancer properties against AGS, HCT116 and HT29 cell lines, yet when given together with CPT-11 or SN38 it affects their therapeutic activity. Our findings shed new light on the application of dietary supplements with antioxidant properties in patients undergoing chemotherapy, indicating the need for further studies on these interactions.

## 4. Materials and Methods

### 4.1. Chemicals

Caffeic acid phenethyl ester (CAPE, 3,4-dihydroxycinnamic acid phenethyl ester, CAS No. 104594-70-9) was purchased from Tokyo Chemical Industry Co., Ltd. (Tokyo, Japan). Irinotecan hydrochloride trihydrate (CPT-11, CAS No. 100286-90-6), 7-Ethyl-10-hydroxycamptothecin (SN38, CAS No. 86639-52-3), DMSO (dimethyl sulfoxide), DAPI (4,6-diamidino-2-phenylindole), MTT [3(4,5-dimethylthiazol-2-yl)-2,5-diphenyltetrazolium bromide], Histopaque 1077, were obtained from Sigma Chemicals Co. (St. Louis, MO, United States). Normal melting point (NMP) agarose and low melting point (LMP) agarose were obtained from Sigma (St. Louis, MO, USA). Penicillin-streptomycin solution stabilized, buffered saline (PBS), fetal bovine serum (FBS), phytohemagglutinin (PHA), RPMI 1640 medium and trypsin-EDTA were supplied by Biowest (CytoGen, Zgierz, Poland). All other chemicals were of the highest commercial grade available.

### 4.2. Cell Culture

#### 4.2.1. Peripheral Blood Lymphocytes (PBLs)

PBLs were separated from leucocyte-buffy coat taken from blood collection in Blood Bank in Lodz, Poland. Blood was gathered from healthy, non-smoking donors of both sexes (aged 20–55) with no signs of infection disease symptoms during collection of the blood. PBLs were isolated using Histopaque 1077 (1.077 g/cm^3^) by centrifugation in a density gradient at 300× *g* for 15 min at 20 °C. PBLs were collected, suspended in the culture medium RPMI 1640, with L-glutamine, 15% inactivated FBS and 1% penicillin and streptomycin. The cells were counted in haemocytometer (Bűrker chamber). The final PBLs density used in the experiments was adjusted to 1 × 10^6^ cells/mL for cytotoxic analysis (MTT test) and 1 × 10^4^ for genotoxic analysis (comet assay) by adding complete RPMI1640 growth medium. Further, 1% PHA was added 24 h before application of the tested compounds to cell suspension. The use of human blood (only leucocyte-buffy coat) in the investigation of the effect of CAPE on human PBLs was approved by Bioethics Committee for Scientific Investigation, University in Lodz (agreement no KBNN-UŁ/1/2015).

#### 4.2.2. Cancer Cell Lines

Three adherent gastrointestinal tumor cell lines: human gastric adenocarcinoma (AGS, ATCC^®^ CRL-1739™), human colorectal carcinoma (HCT116, ATCC^®^ CCL-247™) and human colorectal adenocarcinoma (HT29, ATCC^®^ HTB-38™) supplied by ATCC (Rockville, USA) were used in all experiments. Adherent cell lines were cultured as a monolayer in RPMI 1640 medium supplemented with 10% FBS and 1% antibiotics (penicillin, streptomycin) under a 100% humidified atmosphere of 5% CO_2_ and 95% air, at 37 °C. Additionally, RPMI 1649 growth medium for HT29 was supplemented with 1% amino acids (MEM Non-essential Amino Acid Solution). Exponential growth of cells was maintained by their regular passaging at 90% confluence three times a week using 0.025% trypsin/EDTA.

### 4.3. Cytotoxicity

The MTT assay was used to evaluated cell viability treated with the tested compound. It is a quantitative method based on the tetrazolium yellow dye MTT [3(4,5-dimethyl-2-thiazolyl)-2,5-diphenyl-2H-tetrazolium bromide], which is conversed by living cells to a purple product, formazan, which the concentration was measured colorimetrically. The MTT assay was performed according to the procedure described by Mosmann [56] with a slight modification. Cells were cultured in 96-well microplates (Nunc Brandt Products) at 6 × 10^3^/well in 100 µL appropriate medium for 24 h, then incubated with different concentration with tested compounds for 72 h. After that, 20 µL fresh MTT solution in concentration 5 mg/mL (dissolve in sterile PBS) were pipetted to each well for next 4 h. Subsequently, the medium from the plates with cancer cell lines was removed. To dissolved purple formazan crystals, DMSO (100 µL) was used. In case of lymphocytes, 20%SDS/50%DMF was added (100 µL/well) during the next 24 h. Absorbance was measured with a PowerWave XS spectrophotometer (BioTek Instruments, Inc., Winooski, VT, United States). Cytotoxicity of the tested compounds was evaluated on the basis of their IC_50_ concentrations that reduced cell viability by 50% compared with untreated control cells (cells in medium, not exposed to tested compounds), arbitrary taken as 100%. The IC_50_ values were estimated on the basis of plotting of cell survival (%) curves using GraphPad Prism 7 software [57,58,59]. All results were presented as the means ± SEM of the replicates from six independent experiments.

#### Synergistic or Antagonistic Effect

Synergistic effect means that the final effect of acting of two drugs being co-treated is greater than the sum of the two separate effect, while antagonistic effects is the opposite effect to synergy, and it means the effect of two chemicals causes a decrease of the sum of the effect of the two drug separately. To determine synergistic or antagonistic effect we calculate the ratio (ne/no) of expected cells number (ne) to observed cells number (no) according by Lin et al. [39]. Factor >1 represents synergy effect, while factor <1 represents antagonistic effect CAPE on drug and its metabolite. The MTT test was used to assess the synergistic/antagonistic effect of the test compounds. However, in this case tested compounds were simultaneously administered to the medium for 72 h.

### 4.4. Measurement of Caspase-3/7 Activity

The ability of our test compound to induce apoptosis was determined using the multiplex analytical system ApoTox-Glo™ Triplex Assay. Cell viability was assessed using a fluorogenic peptide substrate (GF-AFC) freely penetrating intact cell membranes of living cells. Inside the cell, the substrate is exposed to cellular proteases, which results in the hydrolysis of peptide bonds and the fluorescent signal emission, which is directly proportional to the number of living cells. In the second step, the DEVD-containing substrate, conjugated with aminoluciferin was used to generate luminescent signals. In this assay DEVD sequences, which are recognized by caspases become cleaved. The resultant cleavage leads to aminoluciferin release and luminescent signals emission due to reaction of aminoluciferin with luciferase. The intensity of emission is directly proportional to the caspase activity in cells treated with the tested compounds. The activity of caspase 3/7 is well established indicator of apoptosis pathway engagement. During apoptosis the cell membrane continuity is maintained (early apoptotic cells), and thus they can be easily distinguished by the first substrate as a viable cells. Testing was carried out in accordance with the protocol provided by Promega. Further, 2 × 10^4^ cells/well were plated 24 h before adding test compounds into a 96-well, black bottom-plate with a transparent bottom. Cells were incubated with tested compounds for 24 and 72 h. After incubating, 20 μL viability/cytotoxicity reagent containing both substrates, GF AFC and bis-AAF-R110, were added to each well. The plate was vigorously mixed (300–500 rpm) for ~30 s and incubated for 1 h in the dark at 37 °C and 5% CO_2_ in air. After incubation, fluorescence was measured at 400Ex/505Em (viability) and 485Ex/520Em (cytotoxicity) using a SpectraMax i3 multifunctional plate reader. In the next step, 100 μL Caspase-Glo^®^ 3/7 reagent was added to the wells. The plate was vigorously mixed (300–500 rpm) for ~30 s and incubated for 1 h at 37 °C in the dark and 5% CO_2_ in air. After incubation, luminescence was measured with the same plate reader.

### 4.5. DNA Damage–Alkaline Comet Assay

Single cell gel electrophoresis (SCGE, comet assay) is a sensitive technique for detection the level of DNA damage. The SCGE was performed under alkaline conditions (pH > 13), according to the procedure of Singh et al. [60] with some modifications as described previously [61]. This version is capable to detect DNA single-strand breaks (SSB), double-strand breaks (DSB), alkali labile sites (ALS), oxidative base damage, and crosslinks with DNA induced by genotoxic agents [62].

#### 4.5.1. Samples Preparation and Lysis

Cells were cultured in 6-well microplates at 1.2 × 10^3^/well in 2 mL medium for 24 h, then incubated with different concentration with tested compounds for 24 h at 37 °C dissolved in complete medium. After incubation, a freshly prepared cells suspension (cancer cells and PBLs) was mixed with 0.75% low-melting (LMP) agarose dissolved in PBS (pH 7.4) and was layered onto microscope slides which were pre-coated with 0.5% normal-melting (NMP) agarose. The slides with cells were stored in the dark for several minutes to allow complete polymerization of the agarose. All next steps were conducted in the dark. After that the coverslips were gently removed and microscope slides were placed for 1 h at 4 °C in lysing solution containing 2.5 M NaCl, 1% Triton X-100, 100 mM EDTA, 1% *N*-lauroylosarcosine sodium, 10 mM of Tris, pH 10.

#### 4.5.2. Electrophoretic Separation and Staining

After lysis, the slides were placed in an electrophoresis unit, and DNA was allowed to unwind for 40 min in the electrophoretic solution consisting of 300 mM NaOH, 1 mM EDTA, pH > 13, at 4 °C. Electrophoresis was conducted at an ambient temperature of 4 °C for 25 min at an electric field strength of 0.86 V/cm, 25 V, 300 mA. After electrophoresis, the slides were placed in a neutralizing buffer containing 0.4 M Tris-HCl (pH 7.5) in the dark for 10 min. The slides were then drained and stained with DAPI (2 μg/mL) for 1 h at 4 °C.

#### 4.5.3. Analysis of Comet Assay

The slides were examined using a fluorescent microscope (Olympus BX 60F5; Olympus Optical Co. Ltd., Tokyo, Japan) equipped with a UV-1 filter block at 360 nm and connected to a computer-based image analysis system, CASP–Comet Assay Software Project by Końca et al. [63]. The head of the comet consists of intact high molecular weight DNA whereas the tail consist of damaged DNA of low molecular weight (single-strand, double-strand breaks or DNA fragments). From each sample were counted minimum of 50 cells and the percentage of DNA in the comet tail (% tail DNA) was measured. The % tail DNA is positively correlated with the level of DNA breakage or/and alkali labile sites in the cell and is negatively correlated with the level of DNA cross-links [64].

### 4.6. Measurement of Reactive Oxygen Species Generation

The measurement of reactive oxygen species generation was done using H_2_DCFDA as described previously by Ruiz-Leal and George [65] with slight modification. H_2_DCFDA, quickly and effectively detect intracellular oxidants. This compound can diffuse through an intact cell membrane and in living cells is hydrolyzed by intracellular esterases to 2′,7′-dichlorodihydrofluorescein (H_2_DCF). Dichlorodihydrofluorescein is oxidized to a fluorescent form, 2′,7′-dichlorofluorescein under the influence of a variety of reactive oxygen species (in our study we used 2 mM H_2_O_2_—determined on the MTT assay results). Tested cancer cell lines (1.2 × 10^4^/200 µL medium) and PBLs (2 × 10^6^/200 µL medium) were seeded into black 96-well culture plates. When they reached 90% confluence, the culture medium was removed (in case of PBLs the plates were previously spun), replaced by 20 mM H_2_DCFDA (dissolve in PBS) and incubated in 37 °C for 20 min prior to addition of compound. Two experimental series were carried out: the first one involved a 30-min preincubation with 2 mM H_2_O_2_, which was used as the oxidative stress inducer; later CAPE (in concentration equal to IC_50_ designated for particular cells) was added at the time of measurement commencement. In the second series, CAPE was added 30 min before the measurement and later 2 mM H_2_O_2_ was applied. Negative control was used in the case of each experimental series (PBS) as well as positive control with CAPE (in the concentration being the IC_50_ values specified for every analysed neoplastic line) and with 2 mM H_2_O_2_ added depending on the experimental series 30 min before the measurement or in the course of measurement commencement. Fluorescence was measured at 15 min intervals for 120 min using an excitation wavelength of 485 nm and emission 535 nm.

### 4.7. Statistical Analysis

Results are presented as a mean ± standard error of the mean (SEM) from three (for comet assay, ROS measurement) and six (for MTT assay) independent experiments. Multiple-group comparisons were carried out using one-way ANOVA with a post-hoc test for subsequent individual group comparison. All statistical calculations were performed using a statistical software STATISTICA (StatSoft Inc., Tulsa, OK, USA). A *p* value of < 0.05 was considered significant.

## 5. Conclusions

This we have found that CAPE had selective cytotoxic and genotoxic activity, directed at neoplastic cells of the gastrointestinal tract, but not at PBLs, which is considerable importance regarding its use in the management. Despite its anticancer activity, CAPE decreases the toxicity of CPT-11 and SN38. We have also demonstrated that CAPE has a protective effect on PBLs against the toxicity of CPT-11 and SN38. This effect may result from the strong antioxidant properties of CAPE, which has been confirmed in our studies.

## Figures and Tables

**Table 1:** Results of analysis of human cancer cell lines and peripheral blood lymphocytes viability after 72 h exposure to CPT-11, SN38 and CAPE at different concentrations compared to untreated control cells.

	IC_50_^a^				
	CAPE	CPT-11	SN38	CAPE + CPT-11	CAPE + SN38
AGS	8.0 ± 1.8 µM	8.0 ± 2.4 µM	0.8 ± 1.6 µM	8 ± 1.7 µM	0.9 ± 0.3 µM
HCT116	8.0 ± 2.1 µM	4.5 ± 1.9 µM	0.5 ± 0. 9 µM	5 ± 2.1 µM	0.5 ± 0.7 µM
HT29	24.0 ± 2.4 µM	8.5 ± 2.9 µM	0.3 ± 1.0µM	8.5 ± 1.1 µM	0.25 ± 0.8 µM
PBLs	ni	200 ± 5.2 µM	16 ± 3.2 µM	ni	ni

^a^ percentage of live cells relatively to the untreated control cells assumed as 100%. ni—no decrease in cell viability; ± SEM.
